# Preclinical ultrasound image-guided high intensity focused ultrasound robot system for breast cancer therapy

**DOI:** 10.1186/2050-5736-3-S1-P42

**Published:** 2015-06-30

**Authors:** Takashi Azuma, Ryusuke Sugiyama, Kazuhiro Matsui, Keisuke Fujiwara, Hideki Takeuchi, Kazunori Itani, Kiyoshi Yoshinaka, Akira Sasaki, Shu Takagi, Ichiro Sakuma, Yoichiro Matsumoto, Toshihide Iwahashi

**Affiliations:** 1 The University of Tokyo, Tokyo, Japan; 2 Hitachi Aloka Medical, Kokubunnji, Japan; 3 National Institute of Advanced Industrial Science and Technology, Tsukuba, Japan

## Background/introduction

Ultrasound imaging provides real-time feedback for highly accurate positioning and dosing control. In addition, spatial restriction of high-intensity focused ultrasound (HIFU) transducer position in the ultrasound image-guided system is less than that in the case of MRI-guided system. Therefore, wider beam approaching path can be used in the ultrasound image-guided system. To shorten the total treatment time, reducing the cooling time between sonication intervals is essential. Using a wide approach path promotes a reduction in both the cooling time and the risk of heat deposition to the body surface. The array in our preclinical HIFU system is supported by a 5-axis robotic system that enables motion with a pivot fixed at the focal point. In this report, we describe a HIFU beam imaging system that provides highly accurate pivoting motion and coagulation monitoring in real-time for dose control during HIFU treatment.

## Methods

A 256-element concave array HIFU operating at 2 MHz was supported by a parallel link robot in a water tank. A 5-MHz linear imaging array was fixed in a hole in the center of the HIFU array. The HIFU beam was affected by acoustic inhomogeneous media. Beam refraction during pivoting motions can cause focal shift and reductions in average focal intensity. We thus developed an HIFU beam imaging method capable of adjusting the focal point in varying approach paths. In the HIFU beam imaging process, backscattered echoes of ultrasound pulses transmitted from the HIFU transducer were received by the imaging array, and an HIFU beam profile was visualized. The imaging array was connected to an ultrasound imaging scanner with a radio frequency (RF) data acquisition system and a ring buffer memory. The ring buffer memory made it possible for the signal processing operations to access the RF data during the acquiring and recording process. Thermal coagulation was able to be detected based on changes in stiffness of the focal tissue. In this prototype system, stiffness change could be monitored based on observation of focal tissue oscillation caused by the modulated HIFU radiation force.

## Results and conclusions

The accuracy of the pivoting motion in our prototype system was evaluated by measuring the precessional radius of the focal point. The motion error was sufficiently-smaller than the width of the HIFU beam. To increase the positioning accuracy of the focal point estimated by the visualized HIFU beam, extraction method of HIFU beam from the distribution of scatter points was tested. For this purpose, the HIFU focal point was scanned and the fixed pattern noise reflecting scatters was strongly suppressed. In coagulation monitoring, the maximum and mean computation times for stiffness estimations from tissue oscillation caused by the acoustic radiation force were 0.85 s and 0.6 s, respectively. To measure tissue oscillation, 26 frames of data were obtained within 0.1 s; therefore, tissue coagulation monitoring with a sampling rate of 1 s was achieved in our prototype system. This sampling speed is considered adequate for enabling feedback control of the coagulation area.

